# Meta-analysis of influences of Biejiajian Pill combined with entecavir on serum liver fibrosis markers of compensatory period of hepatitis b cirrhosis: Protocol of systematic review and meta-analysis

**DOI:** 10.1097/MD.0000000000018458

**Published:** 2019-12-20

**Authors:** Tianyao Zhang, Yu Yang, Baojia Wang, Xiuli Zheng, Long Wang, Xianrong Feng, Guiyu Li, Jinyu Shi, Ning Cao

**Affiliations:** a Chengdu University of Traditional Chinese Medicine; b Hospital of Chengdu University of Traditional Chinese Medicine, Chengdu, Sichuan; c Shaanxi University of Traditional Chinese Medicine, Xianyang, Shaanxi province, China.

**Keywords:** biejiajian pill, entecavir, hepatitis b cirrohsis, liver fibrosis markers in scrum

## Abstract

**Background:**

Chronic viral hepatitis b and its related complications have caused serious harm to human health and become a worldwide public health problem. Hepatitis b cirrhosis is one of the most common complications in Asia. Traditional Chinese medicine combined with antiviral therapy has become the first choice for clinical treatment of hepatitis b Cirrhosis. Biejia Pill is an effective prescription of traditional Chinese medicine in treating Compensatory period of cirrhosis, and there are more and more clinical reports about its validity in treating Compensatory period of cirrhosis. Therefore, we designed this study protocol to evaluate the adjuvant role of Biejia Pill in the treatment of Compensatory period of cirrhosis.

**Method:**

Electronic Databases, PubMed, EMBASE database, Cochrane Library, China National Knowledge Infrastructure (CNKI), Wan Fang, Chinese Scientific Journals Database (VIP) and China Biology Medicine disc, (CBM), will be systematically searched from inception to July 2019. Randomized controlled trials (RCTs) on Biejiajian Pill combined with Entecavir and Entecavir alone against Compensatory period of hepatitis b cirrhosis will be included; inclusion and exclusion criteria will be used to screen the trials. liver fibrosis biomarkers including ECM or its metabolites (serum hyaluronic acid (HA), laminin (LN), procollagen type III (PC-III), and type IV collagen (IV-C)) will be measured as primary outcomes. Liver function, including alanine aminotransferase (ALT) and aspartarte aminotransferase (AST), and improvement of related clinical symptoms will be measured as secondary outcomes. RevMan5 software will be used for literature quality evaluation and data synthesis and analysis.

**Result:**

To evaluate the efficacy and safety of Biejiajian Pill in combination therapy by observing the outcomes of serum liver fibrosis markers, adverse reactions and liver function.

**Conclusion:**

This study protocol will be used to evaluate the efficacy and safety of Biejia Pill in combination with entecavir in the treatment of Compensatory period of hepatitis b cirrhosis, as well as the adjuvant treatment of Biejia Pill in combination.

PROSPERO registration number: CRD42019135402

## 1 Introduction

The main pathological feature of hepatocirrhosis is characterized by fibroblast, accumulation of abnormal muscle fibroblasts, the formation of fibrous scar tissue and extracellular matrix (ECM) protein synthesis and deposition.^[1]^ Liver fibrosis is the early pathological manifestation of cirrhosis, in the early stages, Hepatic fibrosis is reversible.^[2,3]^ However, with the development of the disease and the repair process, self-repair causes self-injury and becomes irreversible, which lead to decompensation of cirrhosis and even the liver cancer. Therefor anti-fibrosis is of great significance in the treatment of liver cirrhosis_,_^[4]^ For hepatitis B cirrhosis, liver fibrosis accompanies with the whole course of chronic hepatitis, and the essence of liver fibrosis is the repair response of the whole body after liver injury, so a single target anti-virus drugs are not ideal in clinical application. The aim of treatment is not only to shorten the HBV replication time, decrease the degree of inflammatory necrosis and fibrosis of liver cells but also to promote the degradation of fiber tissue. Therefore, in clinic, BJJP combined with antiviral drugs have been widely used for the compensatory stage of hepatitis b cirrhosis and have achieved good therapeutic effect.^[5,6,7]^ However, previous meta-analysis did not yet reported the combined treatment for the serum liver fibrosis indicators in the compensatory stage of hepatitis b cirrhosis (the early stage of end-stage liver disease), so we design this research to assess its effectiveness and safety, as well as its adjuvant effects in the combined treatment.

## 2 Method

### 2.1 Registration

This systematic review protocol has been registered on PROSPERO as CRD42019135402. In this paper, the protocol will be performed by the methods introduced in the Cochrane Handbook for Systematic Reviews of Intervention^[8]^ and reported according to the PRISMA-P guidelines.^[9]^ If we will refine procedures described in this protocol, we will document the amendments in the PROSPERO database and disclose them in future publications related to this meta-analysis.

### 2.2 Inclusion criteria

#### 2.2.1 Types of study

According to Cochrane Collaboration's RCT criteria, all references to the words “random sequence ” in the article are regarded as RCTs, regardless of whether they are single-blind, double-blind or non-blind. Languages are limited to Chinese and English.

#### 2.2.2 Study participants

Patients with hepatitis b cirrhosis compensation period, and the 4 indicators of serum liver fibrosis, namely hyaluronic acid (HA), laminin (LN), pre-type-iii collagen (PC III), and type IV collagen (Iv-c), showed at least 3 abnormalities, without the age and sex limitation. Exclusion of decompensation of liver cirrhosis, Non-post-hepatitis cirrhosis, chronic hepatitis with liver cancer, liver failure and other serious cardiovascular, cerebrovascular, endocrine, urinary and hematological diseases; Diagnosis and treatment criteria for hepatic fibrosis Revised by *Guidelines for diagnosis and treatment of liver fibrosis with integrated traditional Chinese and western medicine (2019 edition)*^[10]^; And regardless of age, severity and duration of the disease. Follow-up period is longer than 6 months.

#### 2.2.3 Intervention

Interventions in the experimental group are BJJP combined with entecavir, The interventions in the control group are entecavir. Neither the treatment group nor the control group received additional antiviral and antihepatic fibrosis treatment.

#### 2.2.4 Outcomes

Primary outcomes:

1) the 4 indicators of serum liver fibrosis, namely hyaluronic acid (HA), laminin (LN), pre-type-iii collagen (PC III), and type IV collagen (Iv-c), the outcome were measured by ELISA kit in the beginning and at the end of the treatment course;
2) indicators of Liver function (ALT, AST, DBil), the outcome were measured in the beginning and at the end of the treatment course.

Additional outcomes:

1) HBV-DNA quantification;
2) Adverse reactions: after the start of treatment, the main symptoms of adverse reactions will be recorded, and the number of patients with adverse reactions will be counted.

### 2.3 Search strategy

This meta-analysis will be conducted according to the recommendations of the Preferred Reporting Items for Systematic Reviews and Meta-analyses Statement (Moher et al, 2009). China National Knowledge Internet (CNKI), Chongqing VIP (CQVIP), Wanfang Data, and on-line trial registries such as Clinical Trials. gov (ClinicalTrials.gov/), European Medicines Agency (EMA) (www.ema.europa.eu/ema/), WHO International Clinical Trials Registry Platform (www.who.int/ictrp), PubMed, Embase, Google School, Cochrane Library) will be searched from establishment of the database until May. 2019. We also manually searched additional relevant studies, using references from systematic reviews published previously. The following key words or phrases and their abbreviations or derivatives are utilized singly or in combination:[(“Chinese herbal medicine” or “Chinese medicine” or “traditional Chinese medicine” or “TCM” o*r “*Biejiajian pill*”* or “Biejiajian wan” or “Turtle shells”) and (“entecavir”) and (“hepatitis b cirrhosis” or “liver fibrosis” or “Hepatocirrhosis” or “cirrhosis”) and (“randomized controlled trial” OR “randomized clinical trial”)]. The English language mesh is Biejiajian Pill, cirrohsis, liver fibrosis, the method of combining free words and mesh words to search, and the search items is limited to Chinese and English.

### 2.4 Studies selection

**Figure 1:**
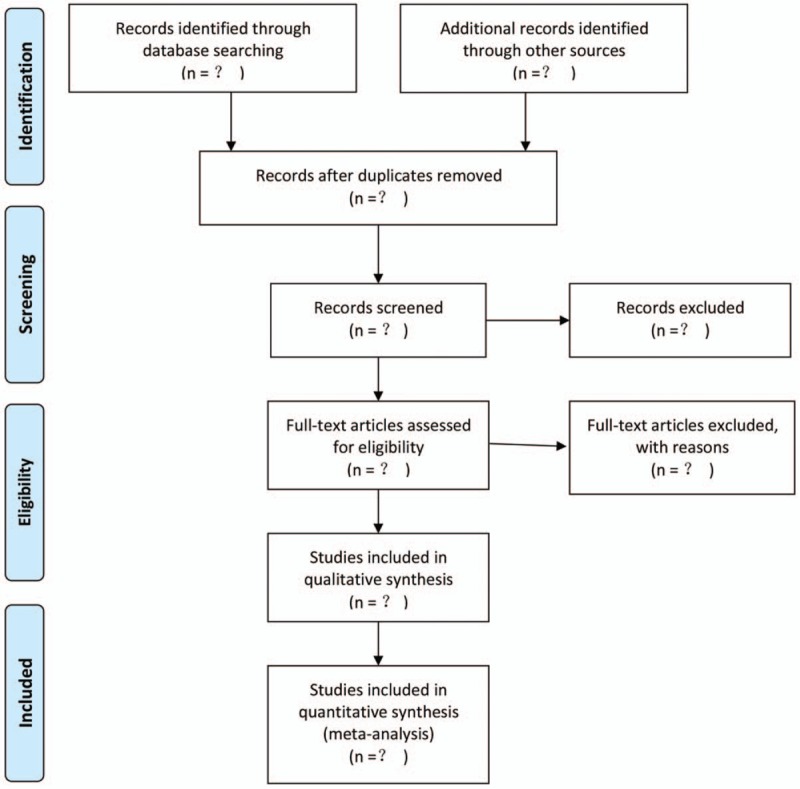
Flow diagram of the study selection process.

Two reviewers (TYZ and BJW) independently screen the titles and abstracts of searching results against prespecified inclusion criteria to identify potential relevance. Disagreements are resolved by consensus. All articles included are judged by the third reviewer (YY). The whole selection process will be presented in a PRISMA flow diagram (Fig. 1).

### 2.5 Data extraction

Two reviewers (TYZ and BJW) will search these databases and independently evaluate all the eligible articles for inclusion. Any disagreement will be resolved through discussion with a third reviewer. Data will be extracted through the predesigned form from the included studies for assessment of study quality and data analysis by 2 individual authors, and any discrepancies will be identified and resolved through discussion with a third author where necessary. If the consensus still cannot be reached, the dispute shall be settled by contacting the original author for original data. Extracted information will include: study population, age, gender and baseline characteristics; details of the intervention and control conditions; follow up time and outcome measures. An excel data extraction form will be devised and piloted in selected studies. Quantitative data for meta-analysis will be extracted on a separate extraction sheet.

### 2.6 Risk of bias (quality) assessment

Two review authors will independently assess the methodological quality of the included trials using The Cochrane Collaboration's tool 5.1. for assessing risk of bias. Domains of the ’Risk of bias’ tool include: Random sequence generation; Allocation concealment; Blinding of participants and personnel; Blinding of outcome assessment; Incomplete outcome data; Selective outcome reporting; Other sources of bias. We will assign a quality rating for the above domains for each included trial as high risk, low risk or uncertain risk of bias. Any disagreements will be analyzed by the third reviewer (YY).

### 2.7 Assessment of publication bias

If more than 10 articles are included, publication bias will be analyzed by visual inspection of funnel plots. A symmetrical distribution of funnel plot data indicates that there is no publication bias.

### 2.8 Strategy for data synthesis

RevMan 5.3.5 software provided by Cochrane collaboration (www.cochrane.org) will be used to conduct meta-analysis and synthesis. Risk ratio (OR) and 95%confidence interval (95% CI) will be used for dichotomous variable; mean difference (MD) and 95% confidence interval (95% CI) will be used for continuous variable; standardized mean difference (SMD) and 95% confidence interval (95% CI) will be used for continuous variable when the units are different. It is considered statistically significant when *P* < .01. Heterogeneity will be assessed using both the χ^2^ test and the I^2^ statistic. When I^2^> 50% or *P* < .10, it indicates that the heterogeneity exceeds the acceptable range, the random effect model will be used. If the heterogeneity is small, in the acceptable range (*P* > .10, I 2 < 50%), the fixed effect model will be used for data analysis. Publication bias will be explored by funnel plot analysis. Publication bias will be explored by funnel plot analysis, and quantitative test of publication bias by Begger method.

### 2.9 Analysis of subgroups or subsets

Subgroup analysis will be based on possible factors that may lead to heterogeneity, such as age, disease course and the HBV DNA levels. If quantitative synthesis is not appropriate, we will conduct a narrative synthesis.

### 2.10 Sensitivity analysis

After subgroup analysis, the heterogeneity between studies remains high, then sensitive analysis will be performed. After excluding a low-quality study, again, meta-analysis is performed to compare the new merger results with the previous ones. If there is no significant change between the two results, the sensitivity would be low; On the contrary, the excluded merger results are quite different from the original merger results or even get the opposite conclusion, then the sensitivity is high.

### 2.11 Confidence in cumulative evidence

GRADE system will be used for assessing the strength of the body of evidence.^[11]^ According to the grading system, the quality of evidence will be rated high, moderate, low, and very low.

### 2.12 Ethics and dissemination

This review is going to be shed in peer-reviewed journals. Our research is a systematic review, which does not contain personal information of patients. Therefore, informed consent and ethical permission are not required for our research.

## 3 Discussion

In the compensatory stage of Hepatitis B Cirrhosis timely and effective treatment could prevent cirrhosis transformation to decompensated cirrhosis and liver cancer. The recognized treatment strategies are antiviral therapy for etiology ^[12]^ and direct antifibrotic therapy for ECM metabolism and HSC activation.^[13]^ Single antiviral therapy cannot inhibit the generation and deposition of liver ECM in fibrotic liver tissues, and single anti-fibrosis therapy cannot inhibit the replication of hepatitis virus and promote the repair of liver injury. Evidence-based medicine (EBM), by integrating individual clinical expertise with the best available clinica evidence from systematic research, indicated that efficiency of antiviral therapy plus TCM can be more effective in longlasting control of hepatitis b cirrhosis than signal therapy,^[14,15]^ BJJW is one of the approved anti-hepatic fibrosis drugs, and has a reliable effect on improving the pathological state of cirrhosis and delaying the formation of liver Fibrosis^[16,17]^; Entecavir is the first choice drug for clinical treatment of hepatitis b cirrhosis, It is guanosine analogue, which mainly inhibits the action of hepatitis b virus polymerase,^[18]^ In recent years, a large number of domestic literatures reported the effect of BJJW combined with entecavir on hepatitis B Cirrhosis.^[19,20]^ In the theory of traditional Chinese medicine, Cirrhosis belongs to the category of “symptoms of lump, accumulation and hypochondriac pain”, BJJW has been used to treat accumulation and lump since thousand years ago, and its efficacy and safety has been testified by long-term practice and evidence-based medicine.^[21]^ Although experimental studies on the treatment of hepatic fibrosis of herbal monomers have made some achievements_,_^[22]^ but the monomers’ cost is more expensive than TCM Compound Prescription. As far as TCM epistemology is concerned, Chinese herbal compound has certain advantages in its pharmacological characteristics of multiple components, multiple levels and multiple targets, which is suitable for the pathogenesis of hepatic fibrosis involving in the whole body after liver injury. Therefor the herbal compound prescription of BJJW has become a hot research for liver fibrosis in recent years.^[23]^ Modern pharmacological studies have shown the mechanism of anti-hepatic fibrosis of BJJW: inhibiting collagen synthesis, promoting extracellular matrix metabolism, inhibiting the release of inflammatory factors, anti-oxidation, improving microcirculation, increasing the degradation of collagen fibers, or directly inhibiting the deposition of collagen.^[24]^ As the main outcome indicators, noninvasive serological markers combined with liver function were the main reference index for diagnosis and prognosis of the liver fibrosis and compensated hepatitis B cirrhosis. Its biomarkers are evaluated by ECM or its metabolites (HA, LN, PC III, Iv-c), the quantification of biomarkers are different in different stages of hepatitis b cirrhosis. Considering that incidence of hepatitis B cirrhosis is closely related to serum HBV DNA level, we set up 2 subgroups in advance for analysis, The one was divided into HBV- DNA ≥ 10^5^ and HBV- DNA ≤ 10^5^_,_ If HBV- DNA ≥ 10^5^_,_ the incidence rate of cirrhosis is higher and the rate of reversion is lower than that of HBV-DNA ≤ 10^5^_;_^[25]^ Another subgroup was classified according to serum AST/ALT ratio, which can sensitively reflect the liver function of patients with hepatitis b cirrhosis.^[26]^ We draft this protocol in order to observe the efficacy and safety of antivirus drug combined with BJJW, and its auxiliary role in combination. This protocol will conduct a meta-analysis of related clinical reports, and provide the current evidence on the efficacy and security of BJJW incombination with Entecavir in treatment of hepatitis b cirrhosis, so as to better guide clinical practice.

## Author contributions

**Conceptualization:** Baojia Wang.

**Data curation:** Xiuli Zheng.

**Formal analysis:** Guiyu Li.

**Investigation:** Long Wang, Xianrong Feng, Guiyu Li, Jinyu Shi, Ning Cao.

**Methodology:** Guiyu Li, Ning Cao.

**Project administration:** Ning Cao.

**Supervision:** Yu Yang.

**Validation:** Yu Yang.

**Writing – original draft:** Baojia Wang.
